# Veterans’ compensation claims beliefs predict timing of PTSD treatment use relative to compensation and pension exam

**DOI:** 10.1371/journal.pone.0209488

**Published:** 2018-12-27

**Authors:** Anne C. Black, Sarah Meshberg-Cohen, Andric C. Perez-Ortiz, Thomas A. Thornhill, Marc I. Rosen

**Affiliations:** 1 Department of Psychiatry, Yale University, New Haven, CT, United States of America; 2 VA Connecticut Healthcare System, West Haven, CT, United States of America; 3 School of Public Health, Yale University, New Haven, CT, United States of America; University of Toronto, CANADA

## Abstract

**Introduction:**

In this study we developed the Disability Beliefs Scale to assess Veterans’ beliefs that engaging in treatment, as well as other behaviors, would affect the likelihood of a Veteran’s being awarded disability-related benefits. We posited that Veterans with stronger beliefs that attending mental health treatment would facilitate a service-connection award would be more likely to attend PTSD treatment before their compensation and pension examinations for PTSD.

**Methods:**

Electronic health records for 307 post-9/11-era Veterans applying for compensation and pension for service-connected PTSD and engaging in a clinical trial of a treatment-referral intervention were analyzed for PTSD-specific and more general mental health treatment use around the time of their compensation examinations. All participants completed the Disability Beliefs Scale and other baseline assessments. Multilevel models assessed change in treatment use as a function of time relative to the C&P exam, compensation examination status (before or after), and the interaction between examination status and beliefs about treatment benefits.

**Results:**

No main effects of time or examination status were observed. As hypothesized, beliefs about treatment benefits moderated the effect of examination status on PTSD treatment use. Veterans believing more strongly that mental health treatment would help a claim differentially attended PTSD treatment before the examination than after. The effect was not observed for general mental health treatment use.

**Conclusion:**

The association between Veterans’ use of PTSD treatment and their service-connection examination status was moderated by beliefs that receiving treatment affects the service-connection decision. This suggests that factors reported to motivate seeking service-connection—finances, validation of Veterans’ experiences, and the involvement of significant others—might also help motivate Veterans’ use of effective PTSD treatments. However, the results reflect correlations that could be explained in other ways, and service-connection was one of many factors impacting PTSD treatment engagement.

## Introduction

Veterans with disabling health conditions that were caused or aggravated by military service may be eligible for disability benefits through the Department of Veterans Affairs [1]. Being designated as having a service-connected health condition confers access to Veterans Health Administration (VHA) services, disability-rated compensation payments and other entitlements. As of 2016, approximately 888,000 Veterans were receiving benefits for service-connected Posttraumatic Stress Disorder [2].

The process of applying for disability benefits involves Veterans’ submitting a claim and undergoing a comprehensive examination by a VA Compensation and Pension (C&P) examiner. Based on both the C&P exam and a review of the Veteran’s VHA health record [3, 4], the examiner submits to the Veterans Benefits Administration (VBA) a semi-structured report (“Disability Benefits Questionnaire for the initial diagnosis of PTSD”) [5] detailing the examiner’s opinion of the diagnosis, severity, and the likelihood that the condition arose as the result of an in-service stressor. In addition to the clinician’s report, the Veteran is encouraged to include with the claim any relevant supporting evidence. VA guidelines explicitly advise Veterans to submit supporting evidence of the claimed condition, including treatment records, *before* the C&P exam [6]. In light of the emphasis on medical records in the claims review process, researchers have asserted that Veterans applying for service-connection pursue VA healthcare services to support their compensation claims [7], and have found greater mental health service use among Veterans after having submitted a claim than before [8]. The co-occurrence of treatment engagement and claim submission might be explained in several ways. Symptoms may drive a compensation claim first and treatment later; symptoms may worsen during the claims process such that treatment is then necessary, or treatment may be sought to support a compensation claim.

VA clinicians have expressed concern that seeking disability compensation for PTSD while attending treatment may have a negative impact on treatment outcomes. Surveyed clinicians rated Veterans applying for service connection for PTSD as less actively engaged in treatment than those who were not seeking compensation [9]. Assessing treatment effects may be more difficult for Veterans with active claims, who may exaggerate their symptom severity during treatment [10]. Despite a highly publicized report of a small arbitrarily-selected cohort of Veterans leaving mental health treatment after being awarded the maximum PTSD benefit [11], a longitudinal cohort study found no such net decrease in treatment use following awarding of disability benefits [12]. Authors of a comprehensive literature review concluded there was no difference in treatment engagement for compensation-seeking and compensation-awarded veterans as compared to non-compensation-seeking veterans [13]. In a recent review of chart data for over 22,000 Veterans with PTSD claims, only 11.5% of Veterans decreased their PTSD treatment utilization after the award decision. The majority of Veterans sustained a pattern of low or no treatment use before and after award, and 9% increased use after award [14].

Understanding Veterans’ beliefs about the relationship between treatment use and disability claims outcomes could provide further insight into individual differences in treatment utilization patterns among Veterans with compensation claims. We posited that differences in patterns of treatment use around the time of the C&P examination would be explained by differences between Veterans in these beliefs. To assess Veterans’ beliefs, we developed the Disability Beliefs Scale (DBS), described below.

In this paper, we first describe the psychometric properties of the DBS for a sample of post-9/11-era Veterans newly seeking compensation for PTSD. We then test the hypothesis that veterans who believe that pursuing mental health treatment increases the likelihood of a Veteran’s being awarded service-connected benefits would be more likely to engage in PTSD treatment specifically in the time preceding their C&P exam than in the time after the exam.

## Methods

The study was approved by the U.S. Department of Veterans Affairs (VA) Central Institutional Review Board. All participants provided written informed consent.

### Sampling and participants

Veterans participating in this study were enrolled in a clinical trial (NCT01597856) testing a brief counseling intervention designed to facilitate engagement in treatment for substance use and/or PTSD. Eligibility criteria for the clinical trial required that participants (a) were post-9/11-era Veterans with a pending initial Compensation and Pension claim for PTSD; (b) were not currently receiving service-connected benefits for PTSD; (c) were able to provide informed consent and participate fully in study procedures; and (d) were between ages 18–65. Recruitment targeted Veterans with scheduled initial PTSD C&P evaluations at VA Connecticut Healthcare System and the Tennessee Valley Healthcare Systems. Letters explaining the research study, including instructions to opt-out, were mailed to 1,951 Veterans. Letters were followed by a phone call from a research assistant. Of the Veterans called, 349 Veterans met study inclusion criteria and completed baseline study assessments with a research assistant. Data were collected between April, 2013 and July, 2016.

Participants from the clinical trial who were included in this secondary analysis had Veteran status during the entire targeted service use period (up to 12 weeks pre- and post-compensation exam) and had complete data on all model predictors. Of the 349 in the clinical trial, 307 Veterans met criteria for this analysis. Sample characteristics are described in Table 1.

**Table 1:** Sample description (n = 307).

Characteristic	N (%) or Mean (SD)
Age	34.27 (8.90)
Male	264 (86.0%)
Race/ethnicity	
Non-Hispanic white	199 (64.8%)
Non-Hispanic black	56 (18.2%)
Hispanic	39 (12.7%)
Other	13 (4.2%)
Married	160 (52.1%)
Separated or Divorced	65 (21.2%)
Single (never married)	82 (26.7%)
Years of Education	14.25 (1.94)
Predominant Work Pattern Past Year	
Regular employment (full or part-time)	143 (46.6%)
Irregular employment	41 (13.4%)
Student	45 (14.7%)
Military service	31 (10.1%)
Not actively working	47 (15.3%)
Days work in past 28 (among 184 employed)	15.39 (9.25)
Any service connection at baseline	116 (37.8%)
Percent service connection (among 116 service-connected)	47.16 (25.31)
Any private insurance	129 (42.0%)
Last month’s income, median (IQR)	$2,650 ($1,400, $4,500)
Years active duty service	7.67 (6.42)
Distance from nearest VA Medical Center, median (IQR)	23.30 (13.20, 48.70)
CAPS Severity Score	61.44 (21.8)
Exposed to clinical trial intervention	80 (26.1%)

### Assessments

All assessments were completed by individual interview with the participant in a private research office. Participants were advised that their responses were confidential and would not be accessible to individuals not affiliated with the research study, and that their participation in the study would not affect their compensation claims or access to VHA health services.

#### Disability beliefs scale

The Disability Beliefs Scale (DBS) was informed by an existing scale measuring attitudes about compensation claims, the Disability Application Appraisal Inventory [15], which includes two items addressing Veterans’ perceiving impacts on their claims. For one item the Veteran rates the impact of “watching what I say” during the compensation exam, and the other involves rating whether a claim “depends entirely on having a doctor or therapist write a letter supporting the claim for service connection.” Other items concerning the relationship between having been in treatment and subsequent receipt of disability payments were adapted from items used in a study of supported employment [16]. Items for the DBS were selected to measure Veterans’ beliefs that the likelihood of service-connected benefits is impacted by Veterans’ (1) seeking treatment for mental health, (2) seeking treatment for substance use, (3) use of drugs/alcohol, and (4) being employed [17].

The DBS asks Veterans to rate 24 items on a 1–5 Likert scale (1 = “much less likely to get benefits”, 5 = “much more likely to get benefits”), indicating how much each proposed situation would affect a Veteran’s chance of getting service-related benefits for the claimed condition (Table 2). For each item, Veterans were asked to rate the likelihood of Compensation and Pension benefits for “*a Veteran*” and not just for themselves specifically.

**Table 2:** Disability beliefs scale items and factor loadings.

Item	F1: Mental Health Treatment	F2: Substance Use	F3: Adaptive Functioning	Decision
1. The veteran is attending mental health treatment regularly	0.801*			Keep F1
2. The veteran takes medication for a psychiatric condition	0.695*			Keep F1
3. The veteran plans to attend mental health treatment	0.612*			Keep F1
4. The veteran occasionally attends mental health treatment	0.585*			Keep F1
5. The veteran uses illicit drugs frequently		0.930*		Keep F2
6. The veteran has a drug use problem.		0.922*		Keep F2
7. The veteran drinks alcohol frequently.		0.897*		Keep F2
8. The veteran has a drinking problem.		0.849*		Keep F2
9. The veteran uses illicit drugs a few times a week.		0.847*		Keep F2
10. The veteran uses illicit drugs occasionally.		0.806*		Keep F2
11. The veteran uses illicit drugs a few times a month.		0.782*		Keep F2
12. The veteran is getting treatment for drug abuse.		0.609*		Keep F2
13. The veteran is working for pay			.472*	Keep F3
14. The veteran is able to work despite symptoms			.452*	Keep F3
15. The veteran drinks a few times a week		0.775*	.434*	Drop, double-load
16. The veteran is having trouble at work		0.639*		Drop, not theoretically consistent
17. The veteran drinks alcohol occasionally		0.482*		Drop, not theoretically consistent
18. The veteran drinks a few times a month		0.474*	.499*	Drop, double-load
19. The veteran is getting treatment for alcohol use	0.537*	0.473*		Drop, double-load
20. The veteran is not employed and not seeking work				Drop, no primary factor loading
21. The veteran’s symptoms make it harder to work				Drop, no primary factor loading
22. The veteran’s substance abuse has stopped with treatment			.678*	Keep F3
23. The veteran does not want mental health treatment			.433*	Drop, not theoretically consistent
24. The veteran’s symptoms have gotten better with treatment			.718*	Keep F3

Loadings < .40 are not shown

Veterans were *not* asked as part of this or any assessment whether they had ever been advised to seek treatment to help their claims.

#### Baseline sample characteristics questionnaire

Participants completed a questionnaire assessing demographic characteristics (age, race, gender, marital status, years education), military experience, number of days worked in the past 28 days, socioeconomic conditions (last year’s work pattern, monthly income by source), percent service-connected benefits for other (non-PTSD) conditions, and access to private insurance.

As an additional measure of access to services, the distance between Veterans’ home addresses and the nearest VA Medical Center were computed to the nearest mile.

#### Clinician-administered PTSD scale for the diagnostic and statistical Manual-IV (CAPS- IV)

Trained research assistants administered the CAPS-IV [18], a widely-used clinical interview designed to derive a continuous index of PTSD symptom severity and diagnosis. Respondents answer questions about the frequency and severity of 17 PTSD symptoms listed in the DSM-IV. PTSD severity scores were computed according to guidelines as the sum of past-month frequency and intensity of each of the 17 symptoms. Severity scores range from 0 (no symptom experience) to 136 (highest frequency and intensity of all symptoms).

#### Treatment utilization

VA treatment for PTSD specifically, and mental health generally, were determined by review of VA electronic health records. PTSD treatment was defined as any outpatient mental health encounter that addressed PTSD or trauma, as determined by chart-specific codes indicating PTSD diagnosis, treatment in a PTSD specialty clinic, and/or clinician notes indicating PTSD was addressed. Mental health treatment was defined using chart-specific codes that indicated an outpatient mental health encounter occurred. For both outcomes, chart-documented events such as research-related treatment, provider chart consultation, and prescription renewal that did not involve patient contact were not counted. The date of Compensation and Pension exam was determined by VHA electronic health record. Treatment received pre-C&P exam was extracted for each week up to 12 weeks before the compensation exam. If the exam occurred fewer than 12 weeks after the claim was filed, service use was only recorded for the weeks from claim submission to exam. Post-C&P exam service use was recorded each week for up to 12 weeks following the C&P exam, or until notification of award, whichever came first.

## Data analysis

To assess the factor structure of DBS items, exploratory factor analysis (EFA) with principal axis factoring was conducted in Mplus software [19], specifying extraction of 2 to 5 factors, and using geomin rotation to allow factors to be correlated. Items were specified as categorical (ordinally scaled) and the solution was estimated using robust weighted least squares. The number of extracted factors was determined by chi-square difference test, model fit indices, and theoretical interpretability of the factor solution. Items were retained if they had factor loadings >.40, no loadings of equal or greater strength on secondary factors, and good theoretical fit with the factor.

Using multilevel modeling with HLM software [20], we then modeled PTSD treatment use as a function of time and C&P exam status. The final model tested the hypothesis that Veterans who believed that pursuing mental health treatment would increase the likelihood of service-connected benefits award for PTSD (according to DBS) were more likely to engage in PTSD treatment within the 12 weeks before the C&P exam than within 12 weeks after the exam. That is, we tested whether the relationship between exam status and PTSD treatment utilization was moderated by beliefs about treatment’s facilitating the service connection claim. Models were estimated using full information maximum likelihood estimation and robust standard errors.

In a secondary analysis, we replicated the final model of PTSD treatment use with the mental health treatment use outcome, testing whether Veterans’ DBS-measured beliefs about mental health treatment similarly moderated the effect of C&P exam status on this more general outcome, or whether the effect of Veterans’ beliefs was specific to patterns of PTSD treatment use.

Model building proceeded in several stages. First, to test whether the probability of PTSD treatment use increased as the exam approached and then decreased afterward, we first specified a piecewise model, estimating unique linear effects of weeks on treatment use within the period before the exam and after the exam. Given no linear change in treatment use within either time period, treatment use was aggregated for the weeks before the C&P exam as having attended any PTSD treatment pre-exam or not, and treatment during the weeks after the exam were similarly aggregated to reflect any post-exam use of PTSD services. Then, the effect of the pre-exam period (relative to post-exam period) on PTSD treatment use was estimated, allowing the effect to vary as a function of DBS-measured beliefs about mental health treatment. The DBS subscale variable was entered using standardized scores to allow interpretation of the effect of the variable in standard deviation (SD) units, give that a 1-point change in raw-score units was equal to 1.79 SD units.

As noted above, although the 12-week periods pre- and post-exam were the focus of the analyses, the number of weeks pre-exam was truncated for some Veterans because their exam occurred fewer than 12 weeks after their claim was filed. Weeks were truncated post-exam if the Veteran received an award decision in fewer than 12 weeks after filing the claim. Because this caused the number of weeks in each period to vary by person, we also tested whether the number of weeks in each period moderated the period’s effect on treatment use.

Measures potentially related to treatment utilization were included in the models as covariates. These variables reflected either access to treatment, treatment-relevant demographics, or need for treatment. The treatment access variables were distance from the nearest VA medical center, days of work in the past 28 days, percent service connection at baseline, having 50% or greater service connection (a threshold at which Veterans are not required to pay anything for medical services), and any private insurance. The treatment-relevant demographic variables were age, gender, race (African-American versus other) and ethnicity (Hispanic versus not Hispanic). Variables reflecting the need for treatment were CAPS-measured severity of PTSD symptoms. In addition, receipt of the clinical trial’s counseling intervention encouraging use of treatment was included as a covariate. Covariates not significantly associated with the outcome were trimmed from the model.

## Results

### DBS factor structure

Exploratory factor analysis revealed a three-factor solution (Table 2). Factors were largely consistent with the expected item structure and fit the data well (RMSEA = .07, CFI = .99, TLI = .99, SRMR = .09). The three-factor solution fit significantly better than the two-factor solution (Δχ^2^(22) = 1210.95, *p* < .001). As expected, factors measured beliefs about the impact on the likelihood of receiving service-connected benefits of mental health treatment (4 items, Cronbach’s alpha = .71) and substance use/treatment for substance use (8 items, Cronbach’s alpha = .94). The third factor, named adaptive functioning, had a broader scope than expected, subsuming items describing both work and improved functioning after treatment (4 items, Cronbach’s alpha = .60). Factor intercorrelations were low: r = .15 (mental health treatment with substance use), r = .04 (mental health treatment with adaptive functioning), and r = -.16 (substance use with adaptive functioning).

Three items were dropped from the instrument because they were not theoretically consistent with the scope of the factors. One item, “The veteran is having trouble at work” was expected to load negatively on a work factor, but loaded on the substance use factor, and was removed because it included no mention of substance use. A second item that loaded on the substance use factor, “The veteran drinks alcohol occasionally”, was dropped because it was not consistent with other items on the factor describing excessive or problematic use. An item loading on the adaptive functioning factor, “The veteran does not want mental health treatment” was dropped because its interpretation was ambiguous, and did not fit theoretically with other items more clearly assessing the effect of improved or adaptive functioning on service connection. Three additional items were dropped due to double-loading, and two others were dropped for not having a single factor loading of at least .40.

The DBS mental health treatment subscale was the focus of these analyses. Scores for this subscale (i.e., the mean of the four-item subscale) were normally distributed around a mean of 3.75/5 (SD = 0.56). Scores within this subscale ranged from 1.75 to 5.0, but 98% of scores were at or above the neutral point of 3.0, reflecting beliefs among most participants that treatment utilization increases the likelihood of service-connection award.

### Treatment utilization

The mean number of weeks available for treatment use after the claim was filed and before the C&P exam (i.e., the pre-exam period) was 10.01 (SD = 2.83, range = 1–12) and after the C&P exam but before the claims decision (i.e., the post-exam period) was 7.29 (SD = 4.20, range = 1–12).

#### PTSD treatment use

**Fig 1:**
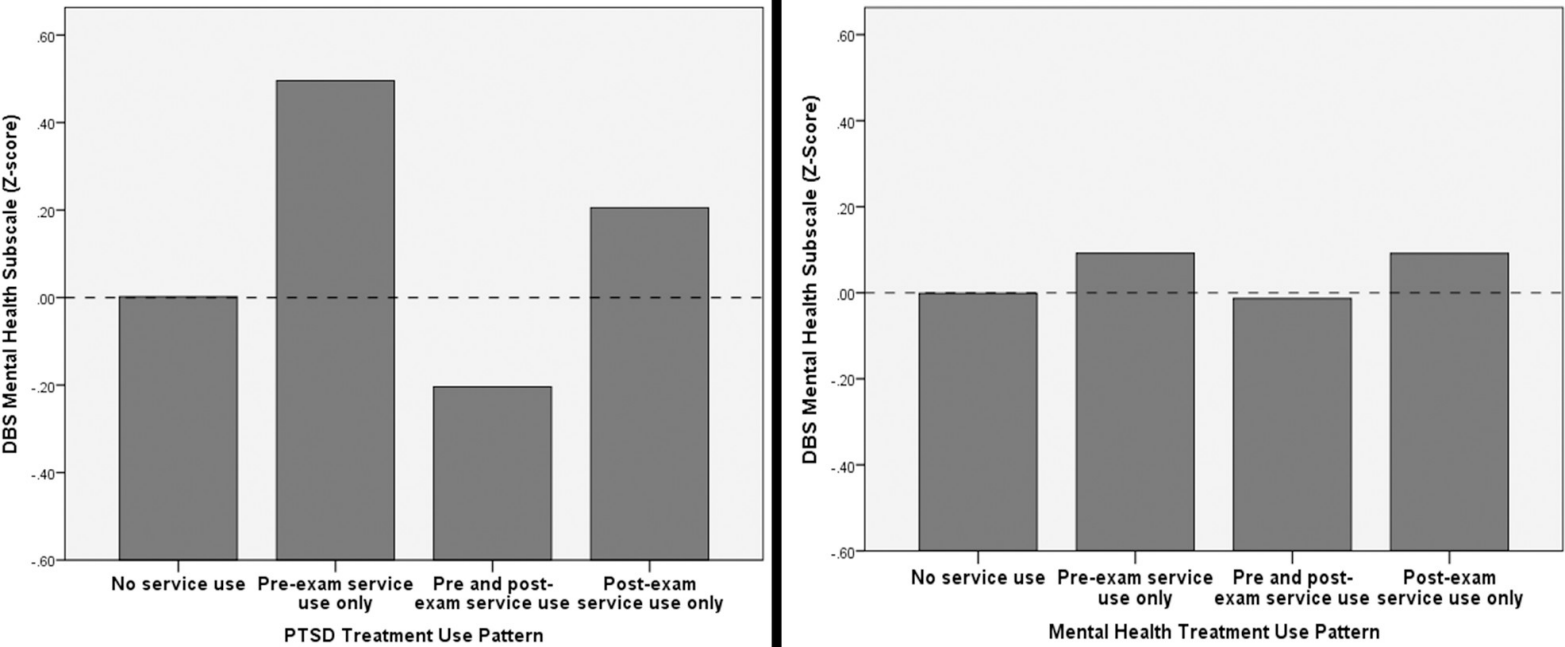
PTSD service use and mental health service use patterns by DBS mental health subscale score.

A total of 51 (16.6%) Veterans used PTSD treatment during the target period. Of those, 15/51 (29.4%) used treatment before the C&P exam exclusively, 12/51 (23.5%) used treatment after the C&P exam exclusively, and 23 (47.1%) used both pre-and post-C&P exam. Fig 1 (left side) illustrates DBS mental health subscale scores (standardized as Z-scores) by treatment use pattern.

In the multilevel model of PTSD treatment use, the effect of the pre-exam period on PTSD service use was not significantly different from zero, indicating the odds of using PTSD treatment before the C&P exam did not differ, on average, from the odds of using PTSD treatment post-exam.

The effect of being pre-exam on treatment use was not significantly moderated by the number of weeks available during the pre-exam period. However, the number of weeks available in the post-exam period was *negatively* associated with treatment use such that Veterans whose award decisions came more quickly after the exam were more likely to have used treatment post-exam. This likely reflects the fact that Veterans whose decisions were made quickly were those with obvious, severe PTSD. Veterans with more severe PTSD having more rapidly adjudicated claims was supported by a post-hoc analysis showing a trend toward a negative correlation between CAPS-measured PTSD severity and number of weeks post-exam available for analysis (*r* = -.10, *p* < .10).

As hypothesized, DBS-measured attitudes about mental health treatment did significantly moderate the effect of being in the pre-exam period on PTSD treatment use. Veterans with higher scores on the mental health treatment subscale of the DBS were significantly more likely to use PTSD services pre-exam (Table 3). An increase of one SD unit on the DBS subscale (equivalent to a raw-score increase of 0.56 points on the 5-point scale) was associated with 34% greater odds of attending PTSD treatment pre-exam than post-exam, relative to the odds for a Veteran with a subscale mean one SD lower. This result was maintained after controlling for effects of covariates on the overall probability of PTSD service use.

**Table 3:** Multilevel logistic regression of PTSD service use.

Fixed Effect	Coefficient	SE	*p*-value	OR	OR95% CI
Intercept *β*_*00*_	-3.00	0.55	<0.01	0.05	0.02, 0.15
Site (TN) *β*_*01*_	-1.81	0.49	<0.01	0.16	0.06, 0.43
Z-DBS MH *β*_*02*_	-0.10	0.20	0.61	0.90	0.61, 1.34
Age *β*_*03*_	0.04	0.02	0.02	1.05	1.01, 1.08
Pct Service Connection *β*_*04*_	0.01	0.01	0.03	1.01	1.00, 1.03
Private Insurance *β*_*05*_	-0.68	0.37	0.07	0.51	0.25, 1.05
CAPS-IV *β*_*06*_	0.02	0.01	0.02	1.02	1.00, 1.04
Wks Post-exam *β*_*07*_	-0.10	0.05	0.03	0.90	0.82, 0.99
Pre-Exam *β*_*10*_	-0.07	0.24	0.77	0.93	0.59, 1.49
Z-DBS MH *β*_*11*_	0.29	0.14	0.04	1.34	1.01, 1.76

#### Mental health treatment use

A total of 156 (50.8%) Veterans used any mental health services at least once during the target period. Of those 43/156 (27.6%) used services in the period before the C&P exam exclusively and 31/156 (19.9%) used services in the period after the C&P exam exclusively (Fig 1, right side).

In the multilevel model of mental health treatment use, there was no significant effect of the pre-exam period. The effect of being in the pre-exam period was not significantly moderated by the number of weeks in the period, nor by DBS-measured attitudes about mental health treatment. This lack of replication provided support for a PTSD treatment-specific effect of DBS-measured attitudes (Table 4).

**Table 4:** Multilevel logistic regression of mental health service use.

Fixed Effect	Coefficient	SE	*p*-value	OR	OR95% CI
Intercept *β*_*00*_	-1.85	0.36	<0.01	0.16	0.08, 0.32
Site (TN) *β*_*01*_	-1.19	0.31	<0.01	0.31	0.17, 0.56
Z-DBS MH *β*_*02*_	0.09	0.14	0.52	1.09	0.84, 1.43
Age *β*_*03*_	0.01	0.01	0.40	1.01	0.98, 1.04
Pct Service Connection *β*_*04*_	0.01	0.00	<0.05	1.01	1.00, 1.02
Private Insurance *β*_*05*_	-0.80	0.24	<0.01	0.45	0.28, 0.72
CAPS-IV, *β*_*06*_	0.03	0.01	<0.01	1.03	1.02, 1.04
Wks Post-exam *β*_*07*_	-0.01	0.03	0.66	0.99	0.93, 1.05
Pre-Exam *β*_*10*_	-0.07	0.13	0.58	0.93	0.71, 1.21
Z-DBS MH *β*_*11*_	0.04	0.12	0.73	1.04	0.83, 1.31

### Effects of covariates

Most covariates had associations with the VA treatment utilization outcomes as expected. All were interpreted in the context of the full models (i.e., controlling for other covariates in the model). In both PTSD and mental health treatment models, percent service connection at baseline was positively associated with treatment use, and having private insurance (i.e., access to care outside of VA), was negatively associated with (VA) treatment use, although this association was statistically significant only in the mental health treatment model. Greater PTSD symptom severity was consistently associated with greater likelihood of treatment use. Age was positively associated with PTSD treatment use as expected but was not associated with mental health treatment use. Veterans at the Tennessee site were significantly less likely to have used either PTSD or other mental health treatment.

Controlling for other variables, gender, race, ethnicity, having at least 50% service connection at baseline, distance from the VA, days of work, and receiving treatment-related counseling were not significantly associated with treatment use, and were removed.

## Discussion

The observed patterns of treatment use in this sample of Veterans seeking compensation for PTSD were similar to patterns observed by McCarthy and colleagues amongst a VA-wide, PTSD-compensation-seeking sample (14). In both samples, the majority sought no treatment, and subsets showed patterns of treatment drop-off, treatment increase, or sustained treatment use around a target event related to their compensation claim.

The overwhelming majority of Veterans in our sample believed that a Veteran would be as likely, or more likely, to receive service-connected PTSD benefits if the Veteran attended mental health treatment. Variability in beliefs explained differences in treatment use patterns as proposed. Veterans with more positive beliefs about the effect of treatment on benefits award were more likely to use PTSD treatment specifically (and not mental health treatment more generally) during the period preceding their PTSD C&P exam relative to post-exam, as hypothesized.

Veterans’ PTSD service use pattern being associated with beliefs about service connection does not necessarily imply that Veterans who are motivated to have their claims awarded are malingering. To the contrary, PTSD severity measured with the CAPS was associated with both general mental health- and PTSD-focused service use. Veterans endorsed high PTSD symptom severity on CAPS interviews that were confidential research assessments, with no obvious incentive for Veterans to exaggerate symptoms. The mean CAPS severity score of 61.4 in this sample is usually associated with substantial impairment and need for treatment [21]. The CAPS scores being associated with PTSD and mental health service use in our sample is consistent with some [22] but not all [23] prior studies of the association between symptom severity and engagement in PTSD treatment. Our data do not permit conclusions about why some Veterans with pre-exam treatment did not continue to use treatment services post-exam; it is possible that individuals were seen for an initial visit pre-exam and ultimately did not pursue treatment, completed treatment pre-exam, or stopped treatment to avoid further reminders of their past trauma [24]. These possibilities are not at odds with an interpretation that the subgroup was differentially motivated toward pre-exam treatment by their beliefs about treatment’s utility vis a vis the claim.

Supporting the influence of PTSD symptoms on treatment-seeking among Veterans applying for service connection is a prior report in which Veterans’ PTSD symptoms and disability levels were higher around the time of their service-connection examinations than in the months before them [25]. Consistent with an impact of financial considerations, unemployed Veterans had disproportionately larger symptom exacerbations from the months before the examinations to the months around the examinations [25].

CAPS measures of PTSD severity were statistically independent of Veterans’ DBS beliefs about treatment (*r* = -.03), with no suggestion that treatment-supporting beliefs were more prevalent among Veterans with fewer true PTSD symptoms. One explanation of the observed independence between PTSD severity and DBS beliefs in this sample is the limited range of DBS scores. However, it is likely that Veterans developed beliefs about factors affecting service connection in unique ways that were independent of their PTSD development, such as their own or others’ prior experience with compensation claims, interpretation of widely-available advice and guidelines about the disability claims process, or more general personal beliefs about navigating legal processes.

Importantly, although guidelines encourage Veterans to provide supporting evidence of the claimed condition in the form of treatment records at the time of their compensation exam, attending treatment is *not* a required condition for award. Proposed historically by Dr. Sally Satel in testimony to Congress, [26]. such policies are proscribed by current statutes. Policies linking the award or maintenance of service connection to attending treatment raise complicated issues about the purpose of service-connection, claimant autonomy, civil rights, and whether such coerced treatment has sustained, or even temporary, efficacy.

Our study findings have implications for how Veterans might be encouraged to access and engage in PTSD treatment. Despite extensive efforts by the VHA, PTSD-focused treatments have been underutilized by Veterans [27–31]. Interviewed Veterans report many disparate motivations for seeking service connection [32]. Financial renumeration was one reason Veterans cited, but Veterans also sought compensation as a way to validate that they had indeed been harmed by their wartime experience. Veterans also reported filing claims in response to people around them who encouraged it. Addressing the financial, psychological, and social motivations to file disability claims might provide a route by which clinicians can engage and maintain Veterans in PTSD treatment. Further, clinicians may administer the DBS to Veterans in treatment who are applying for compensation as a means to assess and address claims-associated motivation for treatment, and erroneous beliefs about the impact of treatment on the compensation award[33].

Alternative interpretations of the study results warrant exploration. It is possible that Veterans’ prior PTSD treatment led them to the belief that treatment and service-connection are linked, rather than causation in the opposite direction. For example, Veterans may learn in PTSD treatment about their PTSD, its impact on their lives, and its origin during military service, and they might conclude that the self-knowledge they obtained during treatment helped their claim. Veterans also might encounter other Veterans while in treatment who pass on their belief that PTSD treatment helped their claim. Another possibility is that the association between beliefs and pre-exam treatment engagement was accounted for by a third variable, such as Veterans having been encouraged to use both VA treatment and compensation services, thus explaining their perceiving a treatment-service connection link, and their simultaneously seeking treatment for PTSD.

It is possible that disability beliefs change over time and as a function of compensation exam status. Thus, having a single measure of beliefs was a limitation. Additionally, chart-documented service use may be an imprecise proxy for actual service use, subject to recording errors. Finally, a small percentage of Veterans in this sample used any PTSD services during the period around their Compensation and Pension exams, and the generalizability of results is limited by this small sample.

## Conclusion

Veterans’ beliefs about the effect of mental health service use on compensation award was associated with more use of PTSD treatment services before the compensation exam, relative to after the exam. Maintaining Veterans in PTSD treatment may be more successful if Veterans’ motivations for service-connection are addressed in ongoing PTSD treatment. Suggestive evidence for addressing compensation-related issues to motivate treatment engagement comes from a study in which Veterans counseled at the time of their service-connection claims for mental health conditions engaged in disproportionately more VA mental health care afterwards[34]. Bundled interventions such as that of Robert Drake and colleagues suggest that altering disincentives to rehabilitation in the disability system can promote better outcomes[35].
